# Predictors for the clinical prognosis of sylvian arachnoid cysts in children

**DOI:** 10.3389/fped.2023.1075087

**Published:** 2023-03-01

**Authors:** Heng Zhao, Wanqun Xie, Liangliang Cao, Zhouwen Ni, Baocheng Wang, Jie Ma

**Affiliations:** Department of Pediatric Neurosurgery, Xin Hua Hospital Affiliated to Shanghai Jiao Tong University School of Medicine, Shanghai, China

**Keywords:** intracranial sylvian arachnoid cysts, prognosis, children, surgery, predictors

## Abstract

**Objectives:**

To investigate the potential factors affecting the clinical prognosis of intracranial sylvian arachnoid cysts(IAC) in children.

**Methods:**

All patients with IAC admitted to our department from January, 1, 2015 to December, 31, 2016, were retrospectively reviewed. Patients were grouped based on surgical treatment (surgery cohort vs non-surgery cohort). The clinical and image outcome of the patients were followed routinely. The clinical characteristics and the prognosis of the patients were compared in different cohorts. Binary logistic regression analysis was applied to analyze the potential factors which may post an influence on the prognosis of the patients.

**Results:**

Of 500 patients admitted to our department for IAC, 424 patients had good prognosis and 76 had poor prognosis, with no deaths occurred during the follow-ups. 68 patients had IAC related complications and 91 patients developed new symptoms during the follow-ups. There were significant differences (*P *< 0.05) between the 2 cohorts in below aspects: age, gender, Galassi subtype, whether the mother was a unipara, the maximum diameter of the cysts at the first visit and the last follow-up, headache, head circumference, temporal bulge, new symptoms, cysts rupture and hemorrhage, subdural effusion, and IAC disappearance. The mean changes in the maximum diameter of the IAC for the patients were marginally higher for the surgery cohort than for the non-surgery cohort (*P* < 0.01). Binary logistic regression analysis suggested that the number of symptom, no new symptoms during follow-up, surgical treatment, age, maximum diameter of cysts at first diagnosis were independent risk factors affecting the prognosis of patients (*P* < 0.05).

**Conclusions:**

Patients older than 22.5 months, with the maximum diameter of IAC greater than 5.75 cm, who have multiple symptoms, born prematurely, develope new symptoms during the follow-ups and obvious symptoms after trauma need to conduct necessary surgical treatment in time. Patients with complications such as cysts rupture with hemorrhage and subdural effusion will acquire good prognosis after timely surgical treatment. IAC complete disappearance warrants no such important attention for the good prognosis.

## Introduction

IAC is a benign intracranial lesion in children with an overall prevalence of 0.3%–2.6%, which is the most common place among different kinds of pediatric intracranial arachnoid cysts (1, 2).The main surgical treatment options include microscopic fenestration (3, 4), neuroendoscopic fenestration (5, 6) and cystoperitoneal shunt (CP shunt) (7). The CP shunt has been gradually replaced by the former 2 methods for the reason of series complication (8). Nowadays, the standard principle of surgical treatment made by surgeon has not reached a consensus. Previous studies suggest that asymptomatic patients should be followed up regardless of the size of the IAC, which do not need early surgical treatment (2).Other studies put forward the opposite theory that aggressive surgical treatment should be conservatively considered for the association between IAC and symptoms is still not clear (9).The objective of this study is to present the clinical characteristic of the IAC and discuss the potential prognosis factors of children with IAC between the 2 cohorts,which may provide reference for the standardized treatment of children IAC.

## Material and methods

### Clinical materials

**Figure 1:**
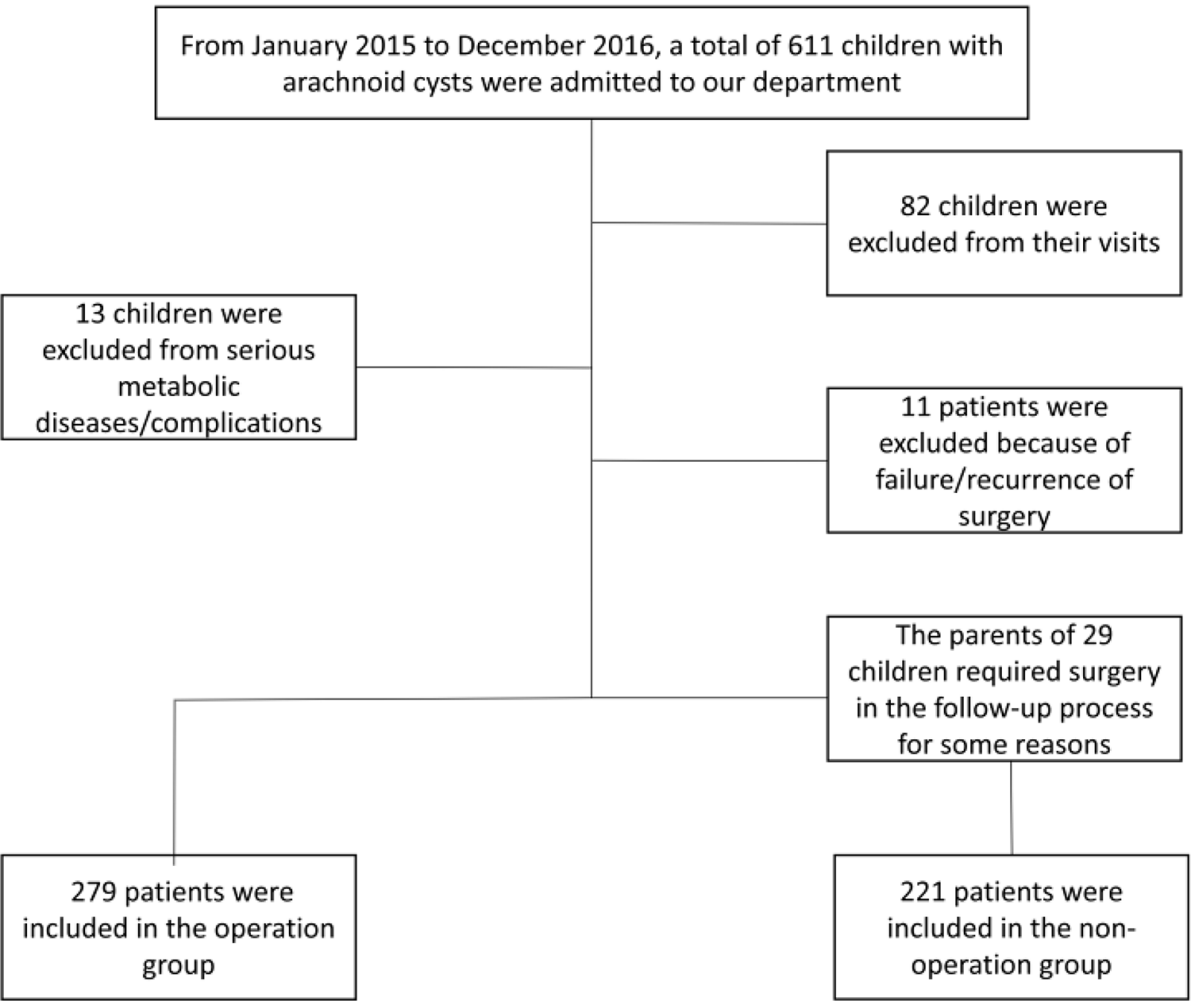
Shows the flow chart for patients with IAC included in this study.

The study population comprised patients who admitted to our department from January 01, 2015, to December 31, 2016, as shown in the Figure 1.

Inclusion criteria: IAC confirmed by preoperative imaging examination; age at first diagnosis ≤18 years;a solitary cyst without concomitant arachnoid cysts in other sites.

Exclusion criteria: comorbidity with other severe metabolic disorders, genetic syndromes and other conditions affecting brain growth and development; incomplete medical record; intracranial hemorrhage history; comorbidity with other intracranial disorders or conditions; recurrence or failure of IAC surgical treatment history.

The data of the patients and their mothers, including: age, gender, birth weight, gestational weeks, whether the children's mothers were primiparas, were obtained through the electronic medical record system and telephone follow-ups.

All children underwent regular head CT or MRI examinations. CT plain scan showed that the IAC were round or round-like homogeneous low-density lesion with clear boundaries, which was the same as the density of cerebrospinal fluid. MRI plain scan showed that the IAC showed long T1 and long T2 signals, which were consistent with the signal of cerebrospinal fluid, and there was no edema in the surrounding brain tissue. IAC enhanced scan showed no enhancement of the wall and contents.

Criteria for good prognosis: (1) The patient's clinical symptoms basically disappear and adapt well to life; (2) There is no intellectual disability related to the development or treatment of IAC; (3) There are no complications associated with IAC or the complications disappear after treatment.; If all the criteria for the good prognosis are not fully met, the prognosis is identified as poor.

The ethical standards followed in this study were in accordance with the World Medical Association Declaration of Helsinki. Patient consent was not required for the retrospective nature of the study.

### Follow-up assessment

Head circumference was measured in children with increased head circumference by taking the circumference from the upper edge of the eyebrow against the scalp to the occipital tuberosity. Then, we would compare the head circumference with the normal range for children of the same age.

Seizures, abnormal behavior, irritability, headache and other symptoms need to be referred to the relevant hospital department, which should be determined by the specialist physician after systematic evaluations.

Developmental retardation need to be evaluated by the child health care physician according to the relevant assessment scales. Subsequently, the patients would be regularly reviewed and evaluated during the follow-ups by the child health care department.

Children with ophthalmic symptoms underwent a comprehensive ophthalmic assessment, including the assessment of eye alignment (strabismus or nystagmus) and examination of the anterior bulbous area and fundus), in order to exclude related eye diseases.

Temporal bone bulge should be examined by physical examination combined with head CT examination, in order to exclude self-related diseases or related syndromes of the temporal bone. Regarding the comparison of IAC during the follow-ups, CT examinations were regularly performed.

Patients with endocrine abnormalities were included in the study after excluding other endocrine diseases and the related endocrine hormone indicators should be regularly monitored for the comparison.

According to the data of image examination, the maximum diameter of cysts were measured at the first visit. Besides, the variations in the maximum diameter of cysts were measured and recorded during the follow-ups. The above monitoring data were routinely evaluated and recorded by 2 pediatric neurosurgeons before and after surgery treatment.

Based on the shape of the cysts and the malformation of adjacent brain structures, the patients were divided into convex and non-convex cohorts (10).

During the follow-ups, subdural effusion, IAC rupture and hemorrhage, hydrocephalus and other conditions were comprehensively evaluated by CT/MRI to determine whether the above complication were related to the IAC.

Tada formula (11) was used to determine the approximate volume of hemorrhage and effusion. Evans index >0.30 (12) was used to confirmed the presence of hydrocephalus.

According to Galassi classification, IAC could be divided into 3 types (13).

Type I: The cysts were small, spindle-shaped and confined to the anterior temporal fossa;
Type II: The cysts are intermediate in size, occupying mainly the anterior middle of the temporal fossa, and may develop along the lateral fissure;
Type III: The cysts are large, occupy essentially the entire middle cranial fossa position, and locally compress the occipital or parietal lobes.

### Statistical analysis

All statistical analysis were conducted by using the SPSS software (version 10.0, SPSS Inc., Chicago, Illinois).Continuous variables with normal distribution were expressed as mean ± 1 standard deviation or median and interquartile range. Comparison between cohorts was analyzed using the independent sample T test. Continuous variables which were not following normal distribution were represented as M (P25, P75), and comparison between cohorts was analyzed using the Mann-Whitney U test. Categorical variables were expressed as the number of cases or percentage composition ratio, and comparison between cohorts was analyzed using the chi-square test or Fisher exact probability. Potential factors showed significant (*P* < 0.15) in univariate analysis and reported significant in clinical practice or previous literature were analyzed by binary logistic regression analysis in which we used the forward stepwise regression method to determine the risk factors which may affect the prognosis of patients. *P* < 0.05 was considered statistically significant.

## Results

### Baseline characteristics

500 patients were enrolled in the study, including 279 patients in the surgery cohort and 221 patients in the non-surgery cohort. The outcomes compromised 400 males and 100 females, with a male to female ratio of 4 : 1. Table 1 presents the basic characteristics of the whole cohort. The average age of the surgery cohort was 56.00 ± 36.33 months, which in the non-surgery cohort was 65.67 ± 39.38 months. Patients aged 1–6 years were divided into a single cohort, and 265 patients of other ages were divided into the other cohort. Of all the Galassi classification IAC patients, there were 277 type II and 223 type III. 335 patients were on the left side and 165 cases were on the right side. Convexity cysts were observed in 311 patients and non-convexity cysts were observed in 189 patients.

**Table 1:** Baseline characteristics.

Baseline Characteristics	Surgery	No-surgery	*P* value
Age (Mean ± SD)	56.00 ± 36.33	65.67 ± 39.38	0.003
Age (*n*, %)			<0.001
1–6 years	156 (55.91)	79 (35.74)	
Others	123 (44.09)	142 (64.26)	
Sex (*n*, %)			0.032
Female	46 (16.49)	54 (24.43)	
Male	233 (83.51)	167 (75.57)	
Galassi (*n*, %)			0.015
2	141 (50.54)	136 (61.54)	
3	138 (49.46)	85 (38.46)	
Side (*n*, %)			0.340
Left	192 (68.82)	143 (64.71)	
Right	87 (31.18)	78 (35.29)	
Convex (*n*, %)			0.577
C	177 (63.44)	134 (60.63)	
NC	102 (36.56)	87 (39.37)	
Premature infant (*n*, %)			0.119
Yes	26 (9.31)	31 (14.03)	
No	253 (90.69)	190 (85.97)	
Low-birth weight infant (*n*, %)			0.319
Yes	12 (4.30)	14 (6.33)	
No	267 (95.70)	207 (93.67)	
Unipara (*n*, %)			0.040
Yes	165 (59.14)	151 (68.33)	
No	114 (40.86)	70 (31.67)	
The first visit	6.14 ± 2.48	4.98 ± 1.22	<0.001
The last follow-up	1.79 ± 1.50	4.11 ± 1.72	<0.001
Change	4.00 (3.20–5.10)	0.50 (0.20–1.10)	<0.001
Follow-up time (Mean ± SD)	76.99 ± 7.89	73.06 ± 7.61	–

**Figure 2:**
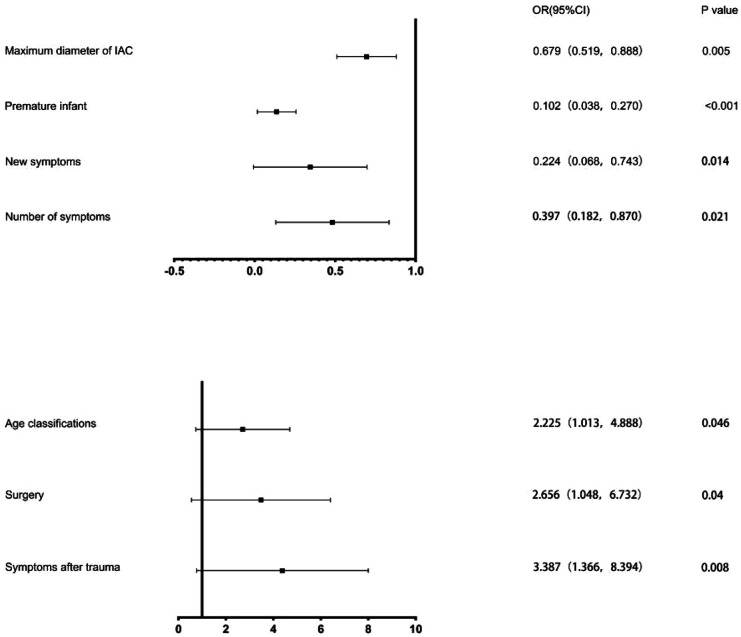
Results of Binary logistic regression analysis. The black squares indicate the OR values, the error bars represent 95% CIs, and *P* < 0.05.

Preterm infants were confirmed in 57 patients, with 26 low birth weight infants and 316 primiparas in the cohort. The maximum diameter of cysts in the surgery cohort and the non-surgery cohort were 6.14 ± 2.48 cm and 4.98 ± 1.22 cm, respectively, and the maximum diameters of cysts at the last follow-up were 1.79 ± 1.50 cm and 4.11 ± 1.72 cm, with the mean follow-up time were 76.99 ± 7.89 months and 73.06 ± 7.61 months, respectively. Significant differences between the 2 cohorts were identified in age, gender, Galassi classification, whether they were premature infants, whether their mothers were primiparas, the maximum diameter of the cysts at the first and last visit, and the variation in the maximum diameter of the cysts (*P* < 0.05). We have not confirmed the significant difference between the 2 cohorts in cysts location (left/right), cysts shape (convexity/non-convexity) and low birth weight (*P* > 0.05). Figure 2 shows the OR value and the *P* value of the above seven factors.

**Figure 3:**
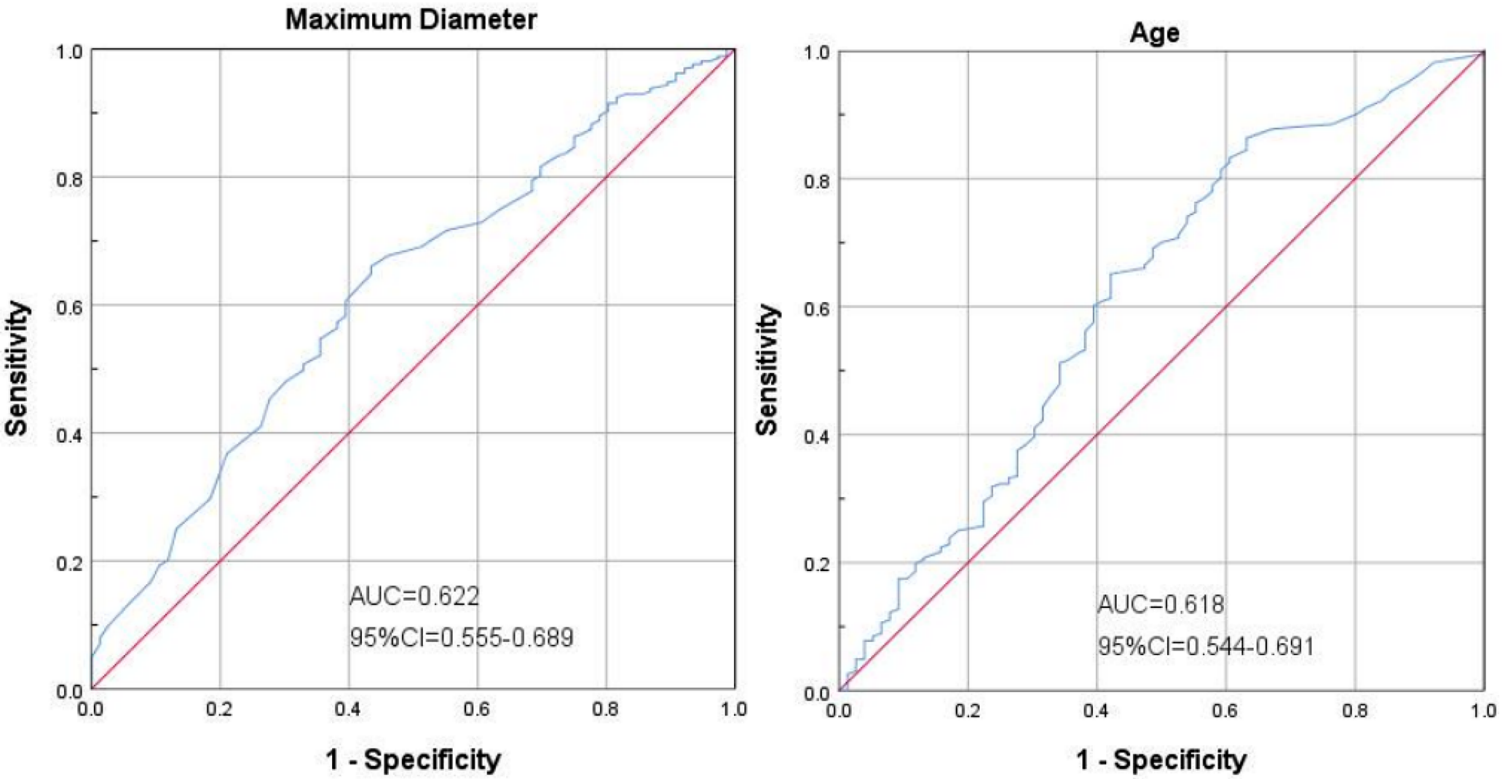
Shows that in the ROC curve analysis, the best sensitivity of age ≥22.5 months to predict the prognosis of children was 86.3%, and the specificity was 63.2%, [area under the curve (AUC) = 0.618; 95%CI: 0.544-0.691]. The maximum diameter of cysts ≥5.75 cm had the best sensitivity of 66.0% and specificity of 56.6% in predicting the prognosis of children [area under the curve (AUC) = 0.622; 95%CI: 0.544-0.691].

Figure 3 shows that in the ROC curve analysis, the best sensitivity of age ≥ 22.5 months to predict the prognosis of children was 86.3%, and the specificity was 63.2%, [area under the curve (AUC) = 0.618; 95%CI: 0.544–0.691]. The maximum diameter of cysts ≥ 5.75 cm had the best sensitivity of 66.0% and specificity of 56.6% in predicting the prognosis of children [area under the curve (AUC) = 0.622; 95%CI: 0.544–0.691].

### Clinical presentation

The most common symptom was headache (221 cases), followed by seizures (90 cases), developmental delay (31 cases), increased head circumference (28 cases), temporal bone protuberances (17 cases), behavioral abnormalities (11 cases), irritability (11 cases), bulging eyeball (6 cases), and endocrine abnormalities (5 cases). 186 children visited the hospital for the reason of head trauma associated symptoms. 248 patients with one symptom were observed and 152 patients with more than one symptom were identified during the entire follow-up period.

30 patients visited the hospital for the reason of IAC rupture, while 38 patients were identified as subdural effusion before the surgical treatment. There were 32 patients who have hydrocephalus before operation and 70 patients whose IAC completely disappeared during the follow-ups.

11 patients with cysts rupture and hemorrhage were included. Patients with a small amount of hemorrhage and no obvious symptoms recovered well after hemostatic treatment. 18 patients underwent surgical evacuation of hematoma and external drainage operation for the reason of obvious symptoms and large amount of hemorrhage, 2 of whom underwent VP shunt due to the persistent symptomatic hydrocephalus after operation, with relieved symptoms at the end.

38 children had subdural effusion, 3 of whom existed obvious symptoms and underwent external drainage operation, with 1 patient underwent subdural-peritoneal shunt operation. The subdural effusion of the other patients disappeared during the follow-ups. Table 2 shows the basic data related to the clinical characteristics of the cohort. The last follow-up of 500 patients manifested that 424 patients had good prognosis, while 76 patients had poor prognosis.

**Table 2:** Clinical presentation.

Clinical presentation	Surgery	Non-surgery	*P* value
Headache (*n*, %)			0.002
Yes	141 (50.54)	80 (36.20)	
No	138 (49.46)	141 (63.80)	
Epileptic seizure (*n*, %)			0.483
Yes	47 (16.85)	43 (19.46)	
No	232 (83.15)	178 (80.54)	
Developmental retardation (*n*, %)			1.000
Yes	17 (6.09)	14 (6.33)	
No	262 (93.91)	207 (93.67)	
Increase head circumference (*n*, %)			<0.001
Yes	25 (8.96)	3 (1.36)	
No	254 (91.04)	218 (98.64)	
Abnormal behavior (*n*, %)			0.123
Yes	9 (3.23)	2 (0.90)	
No	270 (96.77)	219 (99.10)	
Irritability (*n*, %)			1.000
Yes	6 (2.15)	5 (2.26)	
No	273 (97.85)	216 (97.74)	
Eyes bulge (*n*, %)			0.235
Yes	5 (1.79)	1 (0.45)	
No	274 (98.21)	220 (99.55)	
Cryptorrbea (*n*, %)			0.476
Yes	3 (1.08)	5 (2.26)	
No	276 (98.92)	216 (97.74)	
Temporal bone bulge (*n*, %)			0.026
Yes	14 (5.02)	3 (1.36)	
No	265 (94.98)	218 (98.64)	
Symptoms after trauma (*n*, %)			0.113
Yes	95 (34.05)	91 (41.18)	
No	184 (65.95)	130 (58.82)	
Number of symptoms (*n*, %)			0.379
Yes	199 (71.33)	149 (67.42)	
No	80 (28.67)	72 (32.58)	
New symptoms (*n*, %)			<0.001
Yes	83 (29.75)	8 (3.62)	
No	196 (70.25)	213 (96.38)	
Hydrocephalus (*n*, %)			0.716
Yes	19 (6.81)	13 (5.88)	
No	260 (93.19)	208 (94.12)	
Cyst rupture and hemorrhage (*n*, %)			<0.001
Yes	29 (10.39)	1 (0.45)	
No	250 (89.61)	220 (99.55)	
Subdural effusion (*n*, %)			<0.001
Yes	34 (12.19)	4 (1.81)	
No	245 (87.81)	217 (98.19)	
Cyst disappear (*n*, %)			<0.001
Yes	61 (21.86)	9 (4.07)	
No	218 (78.14)	212 (95.93)	
Prognosis (*n*, %)			0.099
Good	230 (82.44)	194 (87.78)	
Poor	49 (17.56)	27 (12.22)	

### Independent factors of a good prognosis

Independent factors associated with a good prognosis were the number of symptoms (1 symptom), disappearance of new symptoms during follow-ups, surgery treatment, age group (1–6 years), and maximum cysts diameter at first diagnosis (the maximum diameter <5 cm) (Table 3).

**Table 3:** Results of Binary logistic regression analysis.

	OR	95%CI	*P* value
Symptoms after trauma	3.387	(1.366, 8.394)	0.008
Number of symptoms	0.397	(0.182, 0.870)	0.021
New symptoms	0.224	(0.068, 0.743)	0.014
Surgery	2.656	(1.048, 6.732)	0.040
Age classifications	2.225	(1.013, 4.888)	0.046
Premature infant	0.102	(0.038, 0.270)	<0.001
Maximum diameter of IAC	0.679	(0.519, 0.888)	0.005

## Discussion

### Dominant results

(1) The maximum diameter of the IAC ≥ 5.75 cm was the best indicator to predict the prognosis, with a sensitivity of 66.0% and a specificity of 56.6%.
(2) The age of patients with IAC ≥ 22.5 months was the best predictor of prognosis, with a sensitivity of 86.3% and a specificity of 63.2%.
(3) The independent potential factors of good prognosis of IAC shows as follow: number of symptoms (1 symptom); no new symptoms during follow-ups; timely surgical treatment;age group (1–6 years old); maximum diameter of IAC at first diagnosis (<5 cm).

Previous literature suggests that when the maximum diameter of type II and III IAC patients are greater than 5 cm (14, 15), the optimal option towards IAC should be surgical treatment. Prevention of IAC rupture is the primary purpose of the above proposal. Our study demonstrated that IAC patients with the maximum diameter greater than 5.75 cm could obtained a good prognosis between the cohorts after timely surgical treatment, in which the difference was significant (*P* < 0.05). Indeed, the maximum diameter of the IAC reflects the effect of the cysts volume. Larger IAC volume reflects more unstable situation, which may have an association with the more obvious symptoms and develop new symptoms. Meantime, the larger size of the IAC, the compression of the brain tissue is severer, which may lead to more cases of developmental delay. In addition, larger IAC may lead to more complication related to IAC rupture, which indicates the poor prognosis. Previous literature reported that subdural hemorrhage accounted for 71.5% of the complications of cysts rupture (16). Our results suggest that patients with lager IAC (the maximum diameter ≥5.75 cm) should be considered to take operation.

0–6 years period lies an important place across the children's brain development, the Karolinska Institute study corroborated the cognitive decline on the neuropsychological tests (17). Research from the School of Medicine at the University of Haukeland in Norway also found that IAC in the dominant hemisphere indeed affected the language development (18, 19). The compression of brain tissue by IAC may lead to the delay or abnormality of brain development, which then cause the developmental and cognitive retardation. We consider the early surgical treatment may lead to a higher frequency of subdural effusion among the patients under 1 year old because the vigorous secretion of arachnoid cysts granules (20).Therefore, in this study, we divided patients aged 1–6 years into a separate cohort. Binary regression analysis showed that surgical treatment performed at the age of 1–6 years could significantly improve the good prognosis. Our study demonstrated that surgical treatment at the patients whose age ≥ 22.5 months was associated with a related better prognosis.

Our results suggest that the maximum diameter of cysts in the non-surgery cohort keeping stable during the follow-ups, which is consistent with the previous literature reported that the volume of most IAC remained stable during the follow-ups (2). The average maximum diameter of IAC at the first visit were significant higher than that at the last visit in the surgery cohort. The results demonstrated that the surgical treatment could achieve ideal results in terms of IAC volume. Furthermore, the complete disappearance ratio of IAC in the surgical cohort is significant greater than that in the non-surgical cohort in the long-term follow-ups (*P* < 0.001),which proves the effectiveness of surgical treatment for eliminating IAC to achieve a good prognosis.

**Figure 4:**
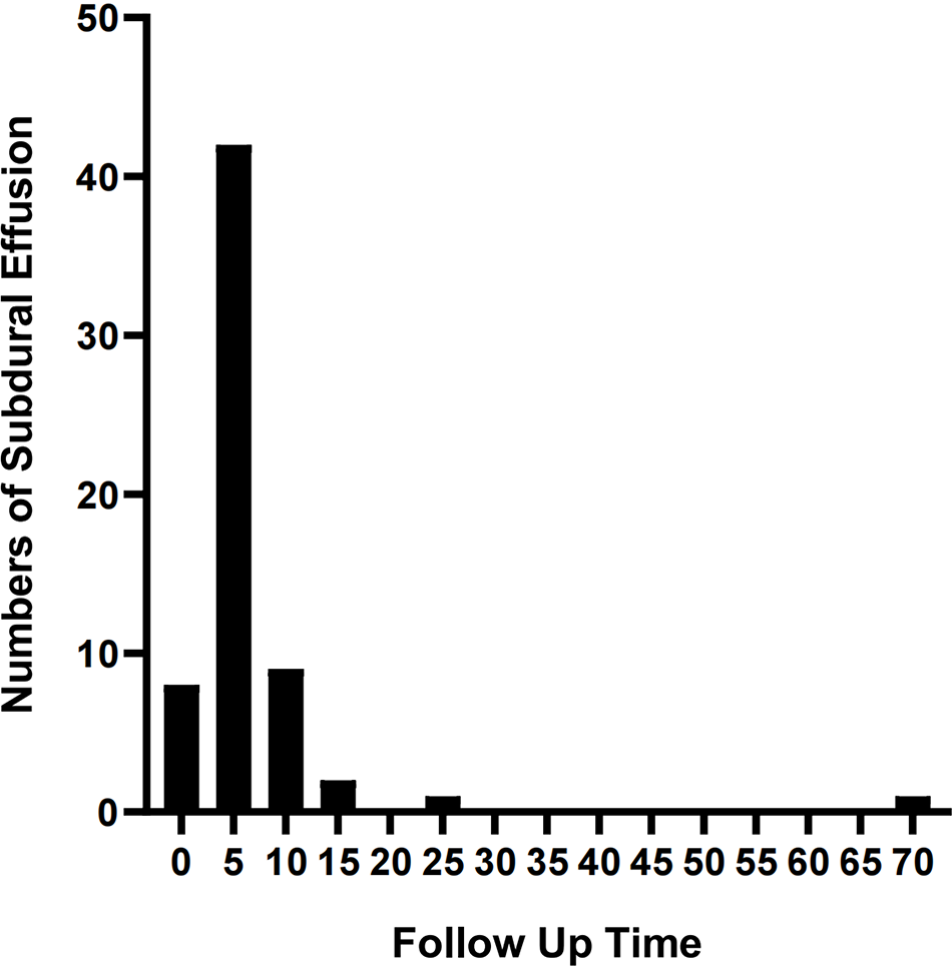
Shows the recovery time of IAC patients with subdural effusion.

**Figure 5:**
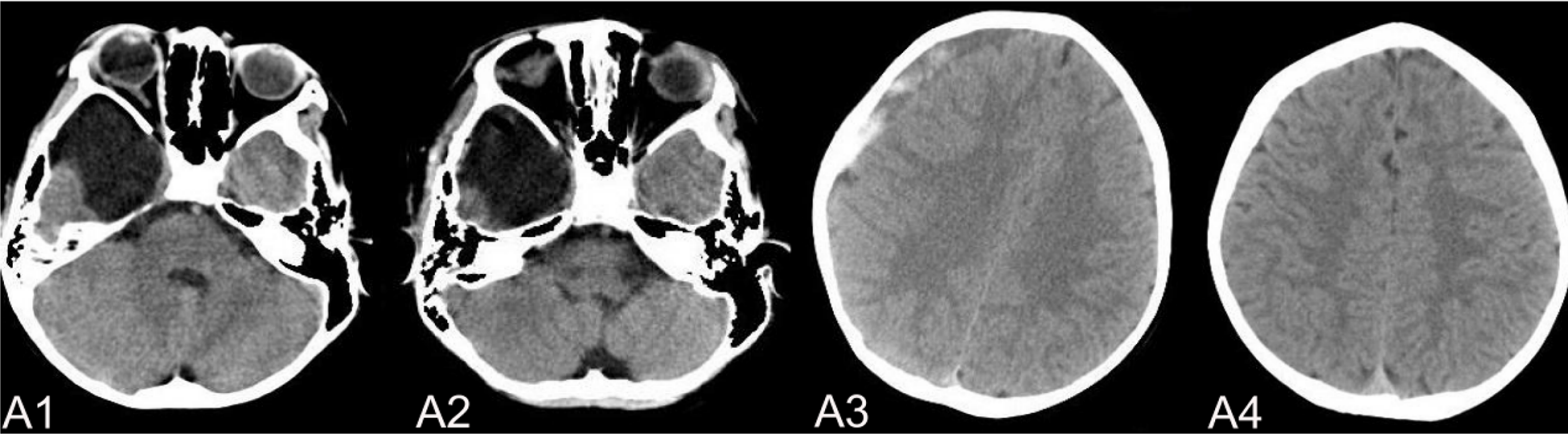
(**A**) shows a patient,who had a subdural hemorrhage during the follow-up time after operation. A1 and A2 shows the IAC CT images before and after operation. A3 shows the IAC hemorrhage after head trauma during the follow-ups. A4 shows the IAC hemorrhage disappeared after hemostasis treatment.

**Figure 6:**
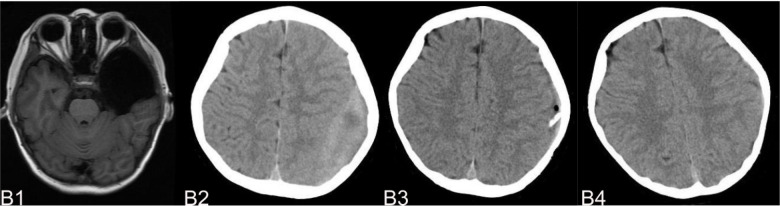
(**B**) shows a typical patient, who had a subdural hemorrhage during the follow-up time after operation. B1 shows the IAC MRI image before operation. B2 demonstrates the IAC hemorrhage after head trauma 2 months after operation. B3 shows the surgical treatment towards the hematoma. B4 shows the recovery of the subdural hemorrhage after the surgical treatment.

**Figure 7:**
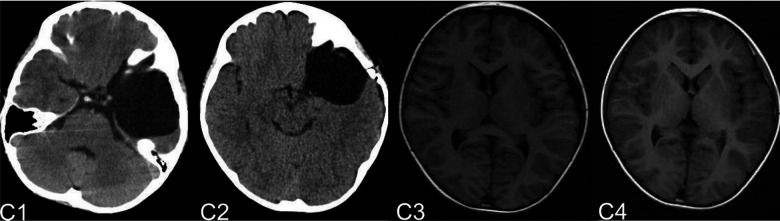
(**C**) shows the patient, who had subdural effusion after IAC operation during the follow-ups. C1 and C2 shows the CT images before and after operation. C3 shows the subdural effusion 2 months after the operation. C4 shows the recovery of the subdural effusion with the treatment of follow up observation after 5 month.

**Figure 8:**
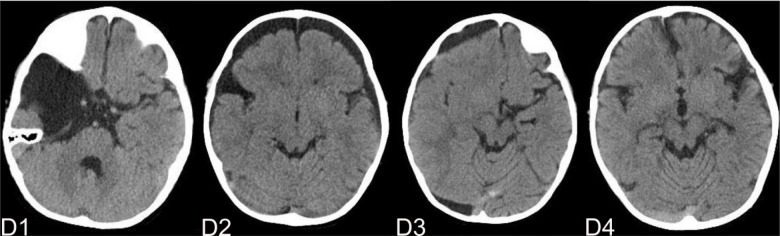
(**D**) shows the patient, who had subdural effusion after IAC operation during the follow-ups. D1 shows the CT image of IAC before operation. C2 shows the subdural effusion 3 month after operation. C3 shows the subdural effusion which decreases after the surgical treatment. C4 shows the disappearance of the subdural effusion 6 months latter.

The complications mainly include IAC rupture and hemorrhage and subdural effusion, which contribute a higher occurrence rate in surgical cohort than that in non-surgical cohort. However, our study demonstrate that cysts rupture and subdural effusion were not independent risk factors for good prognosis in the multivariate analysis. Our study also suggests that temporary cysts rupture and hemorrhage and subdural effusion during the treatment of the IAC, a satisfactory clinical prognosis can still be obtained after timely and reasonable treatment, as shown in the Figures 4–8. As we shows in the Figure 4, of 63 patients with subdural effusion in our study, most of which disappeared in 25 months after operation. Meanwhile, our study also indicate that IAC related complications are mostly associated with head trauma during follow-ups. Therefore, the prevention of head trauma for the IAC patients is warranted to reduce the potential occurrence of complications.

Previous reports showed that most patients with IAC had no obvious symptoms at the first visit of hospital, which were mainly diagnosed accidentally after trauma (2, 21). We included patients who visited our hospital for the reason of head trauma with obvious uncomfortable symptoms in the study. Multivariate analysis showed that patients with uncomfortable symptoms after trauma had a better prognosis than that without uncomfortable symptoms after trauma. The uncomfortable symptoms associated with trauma may indicate the instability of the IAC pressure or the severe compression to the surrounding brain tissue, which will result in significant uncomfortable symptoms in IAC patients even with minor trauma.

Patients who had single symptom or developed no additional new symptoms during follow-ups were independent risk factors about the good prognosis. Multiple symptoms at the first visit or the occurrence of additional symptoms during follow-ups may indicate that the IAC influence on the surrounding brain tissue was grievous and continuous. Thus, our study suggests timely surgical treatment should be considered about the above situation.

Previous reports suggest that the formation of IAC has association with embryonic development (22), so we include the follow risk factors: the birth weight of the child, whether the child was premature, and whether the child's mother was a primipara. Multivariate analysis suggested that preterm birth is an independent risk factor for prognosis (*P* < 0.001).Previous reports point out the differences in the membrane of the arachnoid cysts wall, which can be divided into transparent/thin and pale/tenacious cohorts (23). Preterm birth may affect the composition of the texture of the cysts wall as well ad the compression degree of brain tissue. Therefore, a prospective study of IAC embryo correlation is warranted in future studies to observe more details.

### Limitations

This study was a single-center retrospective study, and the multicenter study were needed in the future. At the same time, we have not systematically studied the prenatal embryological factors and pathogenesis of IAC combined with clinical data. In future studies, the mechanism related to prenatal IAC can be further studied on the basis of prospective study.

## Conclusion

Patient with the below situation: age ≥ 22.5 months, the maximum diameter of IAC ≥ 5.75 cm, multiple symptoms, additional symptoms during the follow-ups, born prematurely, obvious uncomfortable symptoms after trauma, we recommend that the necessary surgical treatment should be performed in time. Complications such as cysts rupture with hemorrhage and subdural effusion during the follow-ups will not have a significant effect on the prognosis of patients after timely treatment. Meanwhile, IAC disappearance is not necessary for good prognosis.

## Data Availability

The raw data supporting the conclusions of this article will be made available by the authors, without undue reservation.
